# Bacterial plasmid-mediated quinolone resistance genes in aquatic environments in China

**DOI:** 10.1038/srep40610

**Published:** 2017-01-17

**Authors:** Lei Yan, Dan Liu, Xin-Hua Wang, Yunkun Wang, Bo Zhang, Mingyu Wang, Hai Xu

**Affiliations:** 1 State Key Laboratory of Microbial Technology, School of Life Sciences, Shandong University, Jinan 250100, China; 2 Shandong Provincial Key Laboratory of Water Pollution Control and Resource Reuse, School of Environmental Science and Engineering, Shandong University, Jinan 250100, China

## Abstract

Emerging antimicrobial resistance is a major threat to human’s health in the 21^st^ century. Understanding and combating this issue requires a full and unbiased assessment of the current status on the prevalence of antimicrobial resistance genes and their correlation with each other and bacterial groups. In aquatic environments that are known reservoirs for antimicrobial resistance genes, we were able to reach this goal on plasmid-mediated quinolone resistance (PMQR) genes that lead to resistance to quinolones and possibly also to the co-emergence of resistance to β-lactams. Novel findings were made that *qepA* and *aac-(6*′)*-Ib* genes that were previously regarded as similarly abundant with *qnr* genes are now dominant among PMQR genes in aquatic environments. Further statistical analysis suggested that the correlation between PMQR and β-lactam resistance genes in the environment is still weak, that the correlations between antimicrobial resistance genes could be weakened by sufficient wastewater treatment, and that the prevalence of PMQR has been implicated in environmental, pathogenic, predatory, anaerobic, and more importantly, human symbiotic bacteria. This work provides a comprehensive analysis of PMQR genes in aquatic environments in Jinan, China, and provides information with which combat with the antimicrobial resistance problem may be fought.

The widespread use of antibiotics since 1930s for the treatment and prevention of bacterial infections both in medical applications and animal husbandry has stimulated bacterial evolution and resulted in the emergence of antimicrobial resistance, which has led to the decrease of the effectiveness of antibiotics^1^. Quinolones serve as a good example of this antibiotic-stimulated emergence of bacterial resistance. The first member of quinolones, nalidixic acid, was introduced in 1962 and was only used occasionally because of its limited effectiveness^2^. Further development of quinolones by creating more efficient fluoroquinolones such as norfloxacin, ciprofloxacin, levofloxacin and moxifloxacin in late 1980s to 1990s has led to more extensive applications of quinolones^3^,^4^. Consequently, while quinolone resistance was rare before 1990, the rate of resistance against fluoroquinolones has increased drastically since its broader application^3^,^5^,^6^.

Bacteria exert resistance against quinolones via primarily two mechanisms: mutations in the chromosomal genes that encode quinolone target enzymes DNA gyrase and DNA topoisomerase IV, regulatory proteins for the overexpression of quinolone efflux pumps, and/or regulatory proteins for the reduced expression of the quinolone in-take system^4^; as well as plasmid-mediate quinolone resistance (PMQR) in which interspecies transferrable plasmid-encoded resistance genes mediate moderate quinolone resistance^7^.

In comparison to mutations in chromosomal genes, the level of quinolone resistance increased by acquiring PMQR genes is low^7^,^8^,^9^. However, PMQR genes do show significant effects on quinolone resistance as was demonstrated in experiments with mouse models^10^; they make a significant supplement to chromosomal mutations on quinolone resistance^9^,^11^; their presence significantly and substantially improved the occurrence of chromosomal mutations^12^,^13^; and their co-existence with extended spectrum β-lactamase (ESBL) genes in hospital samples leads to the phenomenon that selection for quinolones could also result in more severe prevalence of ESBL genes^14^,^15^,^16^. More importantly, PMQR genes can transfer between bacteria at a frequency much higher than chromosomal mutations, making their spread much faster than the occurrence of any other type of antimicrobial resistance. Therefore, understanding the mechanisms by which PMQR genes function and spread, as well as the status of PMQR prevalence in natural, clinical and community settings, is a priority in the combat with resistance against quinolones and other antibiotics such as β-lactams.

Three types of PMQR genes have been discovered: the pentapeptide-encoding *qnr* genes such as *qnrA*^17^, *qnrB*^8^, *qnrS*^18^, *qnrC*^19^, and *qnrD*^20^; efflux pump-encoding genes such as *qepA*^21^; and the *aac-(6*′)*-Ib-cr* gene coding for an aminoglycoside acetyltransferase, a variant of *aac-(6*′)*-Ib*^22^. Surveillance studies have shown these genes are epidemic throughout the world, making the prevalence of PMQR genes an imminent environmental threat^3^,^7^.

To date, the majority of surveillance investigations carried out on PMQR genes used clinical or animal samples, with focus on isolated culturable clinically relevant bacteria such as *Escherichia coli*^23^, *Klebsiella pneumoniae*^14^, and *Enterobacter cloacae*^15^. While this does make sense as antimicrobial resistant pathogens are the primary focus on investigations of antimicrobial resistance genes, these investigations cannot provide information on the full picture of the prevalence of PMQR genes in the biosphere, and cannot help us on assessing the extent and characteristics of the prevalence of PMQR genes in the environment.

Seventy percent of the planet earth is covered by water, making the aquatic environment a key integral part of world’s biosphere. Human activities are heavily dependent upon water systems: people consume water, use water for a variety of human activities, and release part of wastewater resulted from human activities to the aquatic environment. The aquatic environment has been shown a reservoir for PMQR genes^24^, and more specifically, it has been suggested that PMQR genes originated from environmental bacteria such as *Shewanella algae* that primarily inhabit aquatic environments^25^. Therefore, understanding the prevalence of PMQR genes, their correlation between each other and other antimicrobial resistance genes, as well as the distribution of PMQR genes in microorganisms in aquatic systems will give us a good idea on the full picture of the prevalence of PMQR genes in the biosphere.

This work aims to find out the prevalence of PMQR genes in aquatic environments, their correlation with each other, with other antimicrobial resistance genes, as well as their correlation with microbial communities in Jinan, China, using metagenomic and gene quantification analysis approaches. Novel and surprising findings on these aspects were made, and the extent of PMQR prevalence in the environment was assessed.

## Results

### Water samples from four sampling sites in Jinan, China

**Figure 1:**
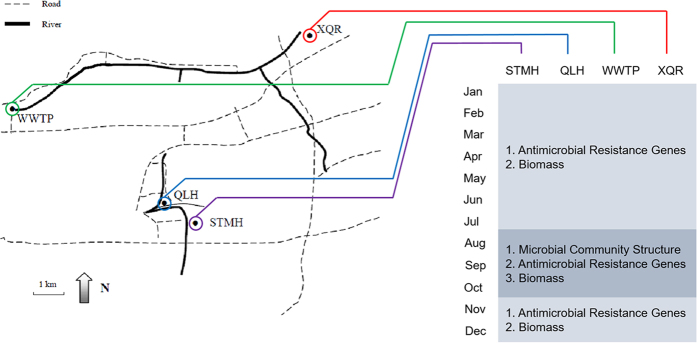
A schematic representation of the geographic locations where water samples were taken, and analyses carried out on these samples.

Water samples were taken for comparison of microbial community structures and antimicrobial resistance gene prevalence from four sampling sites in Jinan, a northeastern city of China, located on the eastern coast of China (36°40′ N, 117°00′ E). These four sites respectively represent a natural water body in a populated city (Xiaoqing River, XQR), water effluent from a urban wastewater treatment facilities (No. 2 Wastewater Treatment Plant of Jinan, WWTP), treated wastewater from a modern hospital (Qilu Hospital, QLH), and treated wastewater from a traditional Chinese medical facility (Shandong Traditional Chinese Medicine Hospital, STMH). The geographic locations of the four sampling sites were shown in Fig. 1.

### Microbial community structures of water samples

**Figure 2:**
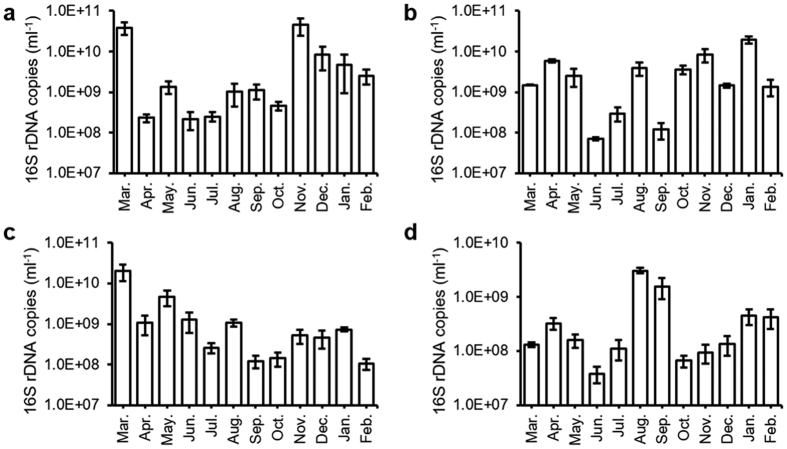
Bacterial biomass levels in collected water samples. Total 16S rDNA levels were used as the measure for bacterial biomass. (**a**) XQR; (**b**) WWTP; (**c**) QLH; (**d**) STMH. Error bar represents standard deviation of six replicates.

Total DNA was extracted from the water samples collected in the four sampling sites monthly from March, 2015 to February, 2016. The level of 16S rDNA abundance was used as a measure for total bacterial biomass. It can be clearly seen that the bacteria content in the wastewater from STMH is lower than all other three water samples (Fig. 2). The year-round average bacterial biomass in STMH samples is 5.49 × 10^8^ 16S rDNA copies/ml, respectively 4.70-, 7.44- and 15.77-fold lower than in QLH (2.59 × 10^9^ 16S rDNA copies/ml), WWTP (4.08 × 10^9^ 16S rDNA copies/ml), and XQR (8.66 × 10^9^ 16S rDNA copie/ml) samples. The observation that XQR has the highest bacterial count suggests wastewater treatment in unban wastewater treatment facilities (WWTP) and hospitals (QLH and STMH) can effectively reduce microbial biomass, while the lower microbial biomass in a traditional Chinese medication facility (STMH) suggests that traditional Chinese medication procedures can effectively inhibit bacterial proliferation.

**Figure 3:**
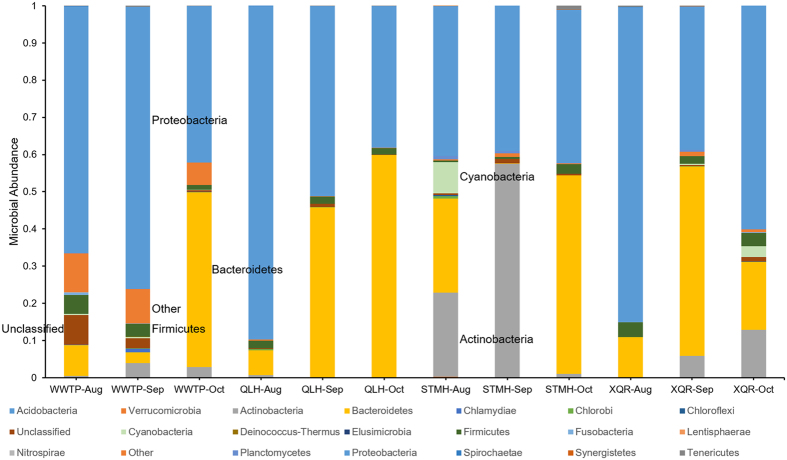
Microbial community structures of the analyzed water samples.

The microbial community structures of water samples collected from the four locations in August, September and October were determined by 16S rDNA high throughput sequencing for comparison (Fig. 3, Table 1). The majority of bacteria in samples taken from QLH and WWTP are Gram negative bacteria from the phyla Proteobacteria and Bacteroidetes, while the Gram positive Actinobacteria are found in significant proportions in STMH and XQR samples. In particular, samples taken from STMH in August and September respectively contain 22.65% and 57.40% bacteria from Actinobacteria. On average, 7.33 phyla take up more than 1% of the total bacteria content in WWTP samples, significantly more than 3 for QLH, 3.33 for STMH, and 4 for XQR (*p* = 2.02 × 10^−4^, 5.82 × 10^−3^ and 7.49 × 10^−3^, respectively), suggesting a more complex microbial community structure in WWTP samples.

### Prevalence of antimicrobial resistance genes in collected water samples

**Figure 4:**
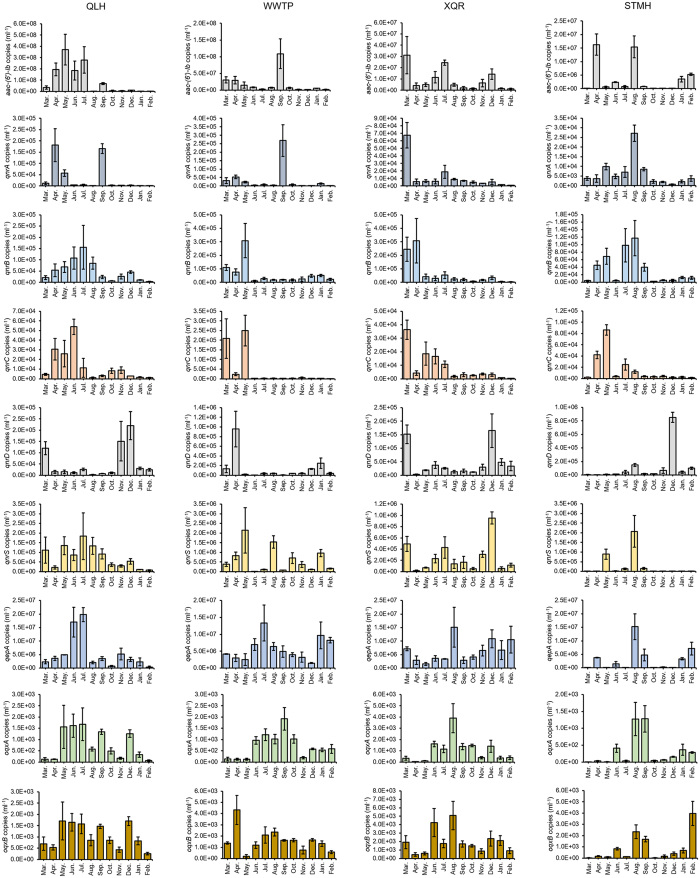
Prevalence of PMQR genes in analyzed water samples. Error bar represents standard deviation of six replicates.

**Figure 5:**
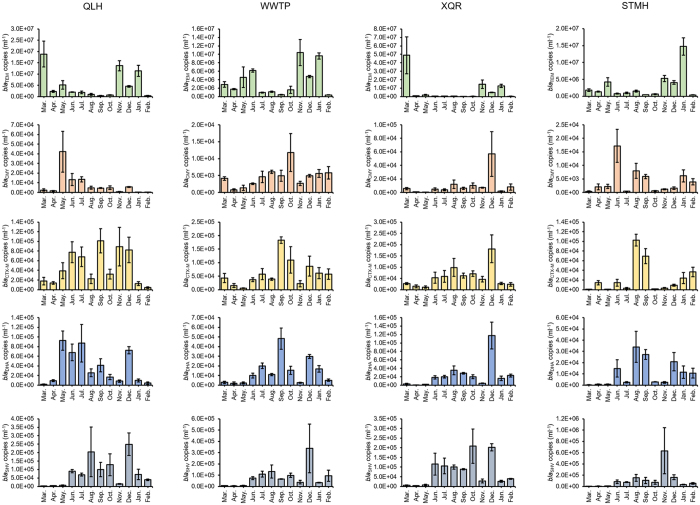
Prevalence of β-lactam resistance genes in analyzed water samples. Error bar represents standard deviation of six replicates.

The prevalence of quinolone resistance genes *qnrA, qnrB, qnrC, qnrD, qnrS, qepA, oqxA, oqxB, aac-(6*′)*-Ib* and the β-lactam resistance genes *bla*_TEM_, *bla*_CMY_, *bla*_CTX-M_, *bla*_DHA_, and *bla*_SHV_ were determined in collected water samples by quantitative PCR (Figs 4 and 5). The levels of these antimicrobial resistance genes show distinct patterns for different genes but similar patterns for the same gene at different locations. A higher level of the β-lactam resistance gene *bla*_TEM_ can be found in winter for all four locations. The levels of other β-lactam resistance genes *bla*_CMY_, *bla*_CTX-M_, *bla*_DHA_, and *bla*_SHV_ are generally low in spring. Higher levels of *qnr* genes were found in spring for nearly every gene at every location. Additional spikes of antimicrobial resistance gene levels could be found for *qnrA* in summer (STMH) and fall (QLH and WWTP), *qnrB* in summer (QLH and STMH), *qnrD* in winter (all four locations), as well as *qnrS* in summer (all four locations) and winter (XQR). The levels of *aac-(6*′)*-Ib* are lower in winter in all four locations. Two spikes on the levels of the efflux pump encoding *qepA, oqxA* and *oqxB* could be generally observed in all four locations: in summer and winter.

Comparison of the levels of antimicrobial resistance genes leads to the finding that although many types of *qnr* genes can be found in water systems, their levels are 2–3 orders of magnitude lower than the efflux pump-coding *qepA* and 3–4 orders of magnitude lower than the aminoglycoside acetyltransferase-coding *aac-(6*′)*-Ib*. The levels of the other two efflux pump-coding genes *oqxA* and *oqxB* are the lowest among all quinolone resistance genes. These findings, together with previous reports that *qepA* provides stronger quinolone resistance^7^, suggest that *qnr* genes do not play a major role in eliciting plasmid-mediated quinolone resistance in the aquatic environments. For β-lactam resistance genes, the level of *bla*_TEM_ is 2–3 orders of magnitude higher than the other genes, suggesting it is the dominant β-lactam resistance gene in the aquatic environments.

Whether antimicrobial resistance genes show similar patterns of abundance is a good indication on whether their expression is co-regulated. Although the *qnr* genes show good agreement in their abundance patterns, the discrepancy in the abundance patterns of *qnr* genes with the other quinolone and β-lactam resistance genes suggests that the abundance of these genes and *qnr* genes is not coordinated. It has been previously shown that different antimicrobial resistant genes may form large operons in mobile elements such as integrons^14^,^26^. These mobile elements can be subsequently transferred between bacteria via horizontal transfer, which may lead to multi-drug resistance in pathogens. The lack of coordination for antimicrobial resistance genes found in this work leads to a relieving suggestion that this issue is not yet widespread in the environments, which leaves room for finding solutions for this potentially devastating problem.

### Correlation between antimicrobial resistance genes

The three most abundant and most important antimicrobial resistance genes found in this work are *qepA* and *aac-(6*′)*-Ib* that are PMQR genes, as well as the β-lactam resistance gene *bla*_TEM_. The analysis of the overall correlation (four sampling sites combined) between these genes showed that the two PMQR genes are significantly correlated but not between *bla*_TEM_ and the PMQR genes (Supplementary Table S1). This is also true for each sampling site except for the natural water sample XQR, in which significant correlation was found between *aac-(6*′)*-Ib* and *bla*_TEM_, but not between the other genes. These findings lead to the suggestion that the coordinated expression of major PMQR genes and β-lactam resistance genes is not yet a widespread phenomenon in aquatic environments, while the major PMQR genes are significantly correlated with each other.

The genes studied in this work other than the three most abundant antimicrobial resistance genes can be generally divided into three groups: the *qnr* genes (the Q group), the efflux pump-coding genes (the P group), and the less abundant β-lactam resistance genes (the B group). When we look at the overall correlation (four sampling sites combined) between the three groups, it can be observed that significant correlation can be observed within the groups but not between the groups, except for that the P group and the B group are significantly correlated (Supplementary Table S1). This observation can lead to the suggestion that genes within the same group show similar functions and they are also similarly regulated. Correlation between *qepA* and the Q group is significantly and strong, also in agreement with this suggestion since they are all efflux pump-coding genes. This suggestion can be also supported by the finding that *qepA*, like the Q group, is also correlated with the B group.

The correlation within each group is also visible when we look at the correlation between genes at each sampling site (Supplementary Table S2). In addition, at each sampling site, more specific correlations were exhibited. In the two hospital samples QLH and STMH, the correlation between group Q and the other two groups, as well as the correlation between group Q, group B and the two major PMQR genes (*aac-(6*′)*-Ib* and *qepA*) became more apparent. This is a suggestion that despite the major PMQR and β-lactam resistance genes remain uncorrelated in the hospital samples; a stronger connection is present between PMQR genes and β-lactam resistance genes in these hospitals. In the natural water sample XQR, the correlation between group B, group Q, *aac-(6*′)*-Ib* and *bla*_TEM_ significantly increased, leading to a worrisome suggestion that PMQR and β-lactam resistance genes, even the major ones, are more significantly correlated in natural water bodies. An overall drastic decrease of correlation between antimicrobial resistance genes could be observed in WWTP that received the highest level of water treatment, which is a suggestion that water treatment can effectively disrupt the correlated expression of antimicrobial resistance genes.

### The correlation between the prevalence of antimicrobial resistance genes and microbial community structures

**Figure 6:**
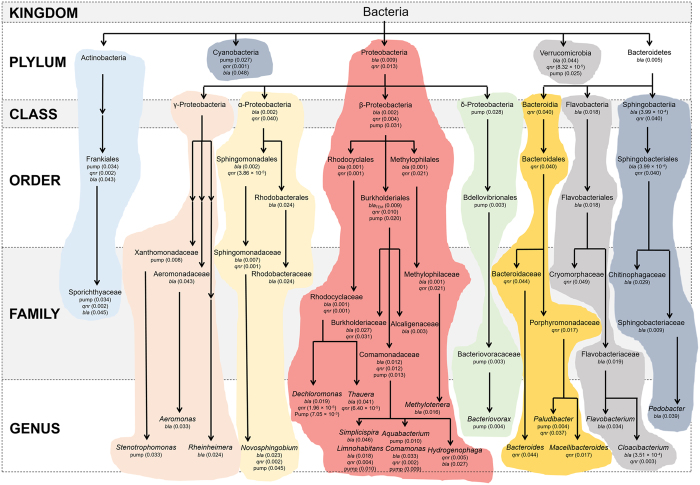
Taxonomic units that are significantly correlated with antimicrobial resistance genes. Under each taxonomic unit are antimicrobial resistance genes that are significantly correlated, followed by *p*-values. *p*-values smaller than 0.05 are considered statistically significant. Pump: efflux-pump coding genes.

The correlation between the microbial community structures in the water samples was analyzed and the prevalence of antimicrobial resistance genes was calculated based on two-tailed Pearson correlation. Significant correlation could be observed for groups of bacteria from Proteobacteria, Bacteroidetes, Actinobacteria, Verrucomicrobia, and Cyanobacteria, particularly in the former two phyla, in which correlation for respective four (α, β, γ, and δ-Proteobacteria) and three (Bacteroidia, Flavobacteria, Sphingobacteriia) classes were found (Fig. 6). Interestingly, no correlation with the prevalence of *aac-(6*′)*-Ib* was found, although it is the most prevalent antimicrobial resistance gene found in this investigation. This possibly indicates the universal prevalence of this gene, to a point where specific association with certain groups of microbes is no long present.

In α-Proteobacteria, significant correlation with *bla* and *qnr* genes was found for Sphingomonadales, in particular the aromatic compound-degrading *Novosphingobium* that may also play a role in pathogenesis^27^,^28^. Significant correlation between Rhodobacteraceae that contain chemotrophic and phototrophic bacteria and the prevalence of *bla* was found^29^.

The β-Proteobacteria appears to have the strongest correlation with the prevalence of antimicrobial resistance genes, with significant correlation with *bla, qnr* and efflux pump-coding genes on the phylum level. The order Rhodocyclales, in particular the family Rhodocyclaceae of this order that contains typically denitrifying and aromatic compound-degrading bacteria such as *Dechloromonas* and *Thauera* spp., is significantly correlated with the prevalence of *bla* and *qnr*^30^. The methylotrophic Methylophilaceae (order Methylophilales) was also found to be significantly correlated with the prevalence of *bla* and *qnr*^31^. The order Burkholderiales of β-Proteobacteria was found to be significantly correlated with *bla, qnr* and efflux pump-coding genes. Two pathogenic families of this order, Burkholderiaceae and Alcaligenaceae, were found to be significantly correlated with the prevalence of *bla* (for both families) and *qnr* (Burkholderiaceae)^32^,^33^. The family Comamonadaceae is significantly correlated with *bla, qnr* and efflux pump-coding genes, in which five environmental genera were found to be correlated with antimicrobial resistance genes: *Comamonas* (correlation with *bla, qnr* and efflux pump-coding genes)^34^, *Limnohabitans* (correlation with *bla, qnr* and efflux pump-coding genes)^35^, *Aquabacterium* (correlation with efflux pump-coding genes)^36^, *Simplicispira* (correlation with *bla*)^37^, and *Hydrogenophaga* (correlation with *qnr* and *bla*)^38^.

No correlation was found between γ-Proteobacteria and antimicrobial resistance genes on the phylum level. However, significant correlation was found between the pathogenic genus *Stenotrophomonas* of the family Xanthomonadaceae and the prevalence of efflux pump-coding genes^39^, the pathogenic genus *Aeromonas* of the family Aeromonadaceae and the prevalence of *bla*^40^, and the environmental genus *Rheinheimera* and the prevalence of *bla*^41^.

The class δ-Proteobacteria was found to be significantly correlated with the prevalence of efflux pump-coding genes. Correlation with efflux pump-coding genes was found in only the order Bdellovibrionales of this class, only the family Bacteriovoracaceae of the order, and only the obligate predatory genus *Bacteriovorax* of the family^42^.

The Bacteroidales order of the class Bacteroidia was found to be significantly correlated with the prevalence of *qnr*. Two groups of these bacteria were found responsible for this correlation: the human gastrointestinal symbiotic genus *Bacteroides* of the family Bacteroidaceae that were found to be significantly correlated with *qnr* prevalence^43^, and the environmental genera *Paludibacter* and *Macellibacteroides* that were found to be significantly correlated with *qnr* (both genera) and efflux pump-coding genes (*Paludibacter* only)^44^,^45^.

The class Flavobacteria was found significantly correlated with the prevalence of *bla*. Two families of the order Flavobacteriales in this class were found significantly correlated with antimicrobial resistance genes: the chemo-organotrophic environmental Cryomorphaceae correlated with *qnr*^46^, and Flavobacteriaceae including the fish pathogenic *Flavobacterium* that is correlated with *bla* and the environmental *Cloacibacterium* that is correlated with both *bla* and *qnr*^47^,^48^.

Two environmental families of the class Spingobacteriia were found to be significantly correlated with the prevalence of *bla*: Sphingobacteriaceae and Chintinophagaceae^49^,^50^, although additional correlation with the prevalence of *qnr* genes was found on the order (Sphingobacteriales) and class (Sphingobacteriia) levels.

## Discussion

The monthly quantification of antimicrobial resistance genes carried out in this work strongly suggests that the outbreak of these genes is seasonal (Figs 4 and 5). The peak of antimicrobial resistance abundance is often more than 2 orders of magnitude higher than the low points of the year. Assuming the distribution of antimicrobial resistance genes in microbes remains generally unchanged throughout the year; this seasonal pattern of a gene is an indication of the seasonal pattern of abundance of the hosts of this gene. If the hosts of two genes are similar and the two genes have correlated expression, their seasonal profiles should be similar. Results shown in this work suggest that seasonal patterns are similar between genes in the same group (B, P and Q groups), but significantly different between genes in different groups, in agreement with statistical analysis results that the abundance of genes in the same group is correlated, while the abundance of genes in different groups are generally not correlated.

Previous research on PMQR genes have suggested that their levels are significantly correlated with other antimicrobial resistance genes, in particular ESBL genes^14^,^15^,^16^. This correlation was particularly significant in clinically relevant bacteria^51^,^52^,^53^. Indeed, plasmids containing both PMQR and ESBL genes controlled by the same promoter has been discovered^54^,^55^. This correlation could lead to the co-selection of quinolone and β-lactam resistance: the extensive use of quinolones on the agricultural sector could lead to the emergence of resistance of β-lactams, which are the most important antibiotics in hospitals.

Results shown in this work suggested that despite the strong correlation of PMQR and ESBL genes in hospitals and food animals, this correlation in the environment is weak: these genes have different seasonal profiles and their abundance lacks statistical correlation, particularly for the most abundant antimicrobial resistance genes. This finding leads to the suggestion that the co-existence and co-selection of resistance to quinolones and β-lactams is not yet pandemic in aquatic environments, and there is still room in fixing this problem.

Three types of PMQR genes are responsible for quinolone resistance: *qnr* genes, *qepA* and *aac-(6*′)*-Ib-cr* that is a variant of *aac-(6*′)*-Ib*^4^. A compiled analysis of surveillance for PMQR genes in over 20,960 isolates before 2008 showed the average prevalence of *qnr* genes and *aac(6*′)*-Ib-cr* is 8.5% and 10.8%^7^. Another surveillance for PMQR genes in 2,297 *E. coli* strains collected from 2004–2011 reported the prevalence of *qnr* genes, *qepA* and *aac(6*′)*-Ib-cr* were respectively 13.6%, 3.3% and 11.6%^56^. The proportions of these three types of PMQR genes were shown to be quite similar. However, results shown in this work showed that the abundance of *qnr* genes fall in the range of 10^3^~10^5 ^copies/ml, several orders of magnitude lower than the *qepA* gene that has an abundance of 10^6^~10^7^ copies/ml and the *aac-(6*′)*-Ib* gene that has an abundance of 10^7^~10^8 ^copies/ml. A substantially higher proportion of *qepA* and *aac-(6*′)*-Ib* was found among PMQR genes. This trend is in agreement with recent clinical surveillance results showing significantly higher occurrence of *aac-(6*′)*-Ib-cr*^56^,^57^,^58^,^59^. Although no comprehensive comparison of PMQR levels in aquatic environments could be found in the literature, two recent investigations on bacteria isolated from aquatic environments suggested the occurrence of *aac-(6*′)*-Ib-cr* is indeed higher than *qnr* and *qepA* genes^60^,^61^. The higher abundance of *qepA* than *qnr* genes reported in this work is on the other hand in agreement with a 2012 report on quantitative comparison of *qnr* and *qepA* genes in swine manure polluted wastewater^62^. Combined, these results suggest a burst of the proportion of *aac-(6*′)*-Ib-cr* and *qepA* among PMQR genes. Considering a similar trend in *aac-(6*′)*-Ib-cr* proportion was found in clinical bacterial isolates, we believe this phenomenon is widespread, rather than limited to aquatic environments.

The more detailed analysis of correlation between antimicrobial resistance genes at the four sampling sites showed varying levels of correlation between PMQR genes and β-lactam resistance genes, although the combined correlation of the four sampling sites is rather weak. In general, a lack of correlation between quinolone and β-lactam resistance can be suggested from statistical analysis. However, a significant correlation between the major PMQR resistance gene *aac-(6*′)*-Ib* and the major β-lactam resistance gene *bla*_TEM_ was observed in XQR, suggesting a stronger correlation between quinolone and β-lactam resistance in the natural aquatic environment, in comparison with wastewater from hospitals and wastewater treatment plants. Particularly, a much weaker correlation between the antimicrobial resistance genes can be observed from WWTP (Supplementary Table S2). These findings lead to an intriguing observation that the rank of correlation between antimicrobial resistance is XQR > QLH/STMH > WWTP. This order is in reverse to the wastewater treatment strength in the four sampling sites: In WWTP, the wastewater was treated by sequentially going through 3 aerobic digesters, 1 anaerobic digester and UV radiation; in hospitals only precipitation and chemical or physical disinfection was performed; and no treatment was performed in natural water bodies. This interesting observation may lead to the suggestion that wastewater treatment can abolish correlation between quinolone and β-lactam resistance.

The correlation between a group of bacteria and antimicrobial resistance genes provides evidence on whether these bacteria may contain these antimicrobial resistance genes, although it needs to be noted that this evidence is not conclusive. It was previously suggested that the aquatic environment is a reservoir for PMQR genes and aquatic bacteria may be the origin for these genes^24^,^25^. Results obtained in this work are in agreement with this finding, by showing that environmental bacteria from families such as Rhodocyclaceae and Cryomorphaceae and genera like *Aquabacterium, Limnohabitans, Comamonas, Paludibacter, Macellibacteroides*, and *Cloacibacterium* are significantly correlated with the PMQR genes. Significant correlation was also found between PMQR genes and pathogenic bacteria from families Sphingomonadaceae and Burkholderiaceae, as well as the genus *Stenotrophomonas*. To our surprise, significant correlation was also found between PMQR genes and obligate predators from *Bacteriovorax*, anaerobic methylotrophic bacteria from *Methylotenera*, and human gastrointestinal symbiotic *Bacteroides* species. These findings lead to the implication of the prevalence of PMQR genes not only in previously better recognized environmental and pathogenic bacteria, but also in less well-known predatory, anaerobic and human symbiotic bacteria. Previous research has already suggested the presence of antimicrobial resistance genes in probiotic bacteria such as lactic acid bacteria^63^. This previously documented occurrence of antimicrobial resistance genes in probiotic bacteria, along with the finding in this work that human symbiotic bacteria may host PMQR genes is disturbing, as antimicrobial resistance gene-hosting probiotic bacteria may do more harm than pathogens.

The use and misuse of antibiotics has led to the emergence of antimicrobial resistance in bacteria, which ranks among the most serious threats to human’s health in this century. To tackle this problem, the current status of antimicrobial resistance prevalence in the environment has to be fully assessed. However, previous research has primarily focused on selected populations and selected bacteria, which can potentially lead to a biased assessment of the current antimicrobial resistance problem. In this work, we aim to use metagenomics and gene quantification approaches to get a comprehensive and unbiased picture of the prevalence of PMQR genes in aquatic environments, which are known reservoirs of PMQR genes and one of the most important parts of the biosphere. In conclusion, we were able to determine the prevalence of PMQR genes, the relative abundance of each gene, the correlation between PMQR genes and other antimicrobial resistance genes, as well as the correlation between these genes with the microbial community structures. Important findings were made in this work with these analyses: (1) The overall correlation between PMQR and β-lactam resistance genes in aquatic environments is not yet strong, providing room for fixing the potential PMQR-ESBL co-selection problem; (2) It appears that efficient wastewater treatment can reduce correlation between antimicrobial resistance genes; (3) the major form of PMQR in aquatic environments are the aminoglycoside acetyltransferase-encoding *aac-(6*′)*-Ib* and the efflux pump-encoding *qepA*; and (4) the prevalence of PMQR genes has been implicated in not only conventional environmental and pathogenic groups of bacteria, but also in predatory, anaerobic and more disturbingly, potentially probiotic bacteria. This work provides the basis for assessment of antimicrobial resistance prevalence, and the finding of solutions to solve this worldwide problem.

## Methods

### Water sampling and total DNA extraction

Surface water samples were taken for investigation from Xiaoqing River, water effluent from No. 2 Wastewater Treatment Plant of Jinan, treated wastewater from Qilu Hospital, and treated wastewater from Shandong Traditional Chinese Medicine Hospital monthly from March 2015 to February 2016, between the 25^th^ and 30^th^ of each month. These water samples were stored on ice upon extraction and processed immediately for downstream experiments.

Five hundred milliliters of water samples were filtered using filter paper to get rid of possible large chunks of solids. The filtered water samples were further filtered using 0.22-μm filters. Total genomic DNA was extracted from these filters using a plant genomic DNA extraction kit (Tiangen Biotech Co., Ltd., Beijing, China). Approximately 100 μl of total genomic DNA was obtained. Three biological replicates for each water sample were carried out.

### Microbial community structure analysis

The 16S rDNA fragments were amplified from each DNA sample and sequenced by high-throughput sequencing for the analysis of microbial communities in water samples taken in August, September and October. Each 16S rDNA sequence was assigned to respective taxonomic groups based on sequence similarities.

### Quantification of 16S rDNA and antimicrobial resistance genes

For quantification of 16S rDNA and antimicrobial resistance genes in each water sample, primers with amplification efficiency between 0.9–1.1 were designed and tested for each analyzed gene (Supplementary Table S3). The quantification of genes in 2 μl of extracted genomic DNA samples subsequently took place in a LightCycler 480 II real-time PCR system (Roche Applied Science, Mannheim, Germany), using SYBR Premix Ex Taq™ (TliRNaseH Plus) (Takara Bio Inc., Shiga, Japan). Six replicates for each reaction were carried out.

### Analysis of correlation

The correlation between antimicrobial resistance genes was assessed using one-tailed Pearson correlation, as only positive correlation between antimicrobial resistance genes has been discovered previously and deemed possible. The correlation between antimicrobial resistant genes and bacterial biomass was assessed by calculating two-tailed Pearson’s correlation between levels of antimicrobial resistance genes and amounts of bacteria (represented by total 16S rDNA content in the sample times the percentage of a bacterium). A *p*-value smaller than 0.05 was considered statistically significant.

## Additional Information

**How to cite this article**: Yan, L. *et al*. Bacterial plasmid-mediated quinolone resistance genes in aquatic environments in China. *Sci. Rep.* **7**, 40610; doi: 10.1038/srep40610 (2017).

**Publisher's note:** Springer Nature remains neutral with regard to jurisdictional claims in published maps and institutional affiliations.

## Supplementary Material

Supplementary Information

Supplementary Table S1

Supplementary Table S2

## Figures and Tables

**Table 1:** Top 3 most abundant microbial groups at each taxonomic level.

	Phylum	Class	Order	Family	Genus
WWTP-Aug	Proteobacteria (66.35%)	Gammaproteobacteria (55.77%)	Pseudomonadales (53.70%)	Moraxellaceae (36.24%)	*Acinetobacter* (36.11%)
	Bacteroidetes (8.22%)	Bacteroidia (7.06%)	Bacteroidales (7.06%)	Pseudomonadaceae (17.46%)	*Pseudomonas* (17.46%)
	Firmicutes (5.01%)	Betaproteobacteria (3.57%)	Campylobacterales (2.04%)	Bacteroidaceae (3.46%)	*Bacteroides* (3.46%)
WWTP-Sep	Proteobacteria (75.67%)	Gammaproteobacteria (60.72%)	Pseudomonadales (47.85%)	Moraxellaceae (41.59%)	*Acinetobacter* (39.64%)
	Actinobacteria (3.91%)	Alphaproteobacteria (10.90%)	Enterobacteriales (11.22%)	Enterobacteriaceae (11.22%)	*Enterobacter* (8.14%)
	Firmicutes (3.91%)	Actinobacteria (2.77%)	Rickettsiales (5.22%)	Pseudomonadaceae (6.26%)	*Pseudomonas* (6.26%)
WWTP-Oct	Bacteroidetes (47.02%)	Flavobacteria (43.40%)	Flavobacteriales (43.40%)	Flavobacteriaceae (43.24%)	*Flavobacterium* (40.12%)
	Proteobacteria (42.07%)	Alphaproteobacteria (15.04%)	Pseudomonadales (10.36%)	Moraxellaceae (7.78%)	*Acinetobacter* (7.73%)
	Actinobacteria (28.80%)	Gammaproteobacteria (13.15%)	Burkholderiales (7.23%)	Caulobacteraceae (7.01%)	*Brevundimonas* (6.76%)
QLH-Aug	Proteobacteria (89.69%)	Gammaproteobacteria (80.67%)	Pseudomonadales (79.67%)	Moraxellaceae (79.39%)	*Acinetobacter* (78.72%)
	Actinobacteria (6.68%)	Betaproteobacteria (7.70%)	Rhodocyclales (5.22%)	Rhodocyclaceae (5.22%)	*Chryseomicrobium* (1.82%)
	Firmicutes (2.04%)	Sphingobacteriia (3.51%)	Sphingobacteriales (3.51%)	Flavobacteriaceae (2.82%)	*Sediminibacterium* (1.17%)
QLH-Sep	Proteobacteria (51.02%)	Flavobacteria (36.98%)	Flavobacteriales (36.98%)	Cryomorphaceae (24.52%)	*Fluviicola* (24.52%)
	Bacteroidetes (45.61%)	Alphaproteobacteria (31.90%)	Caulobacterales (20.84%)	Caulobacteraceae (20.84%)	*Brevundimonas* (19.71%)
	Firmicutes (1.94%)	Betaproteobacteria (8.87%)	Rhizobiales (9.73%)	Flavobacteriaceae (12.46%)	*Chryseobacterium* (10.79%)
QLH-Oct	Bacteroidetes (59.75%)	Flavobacteria (48.68%)	Flavobacteriales (48.68%)	Flavobacteriaceae (46.98%)	*Cloacibacterium* (28.37%)
	Proteobacteria (38.11%)	Epsilonproteobacteria (16.37%)	Campylobacterales (16.37%)	Campylobacteraceae (16.32%)	*Flavobacterium* (18.23%)
	Firmicutes (1.69%)	Gammaproteobacteria (14.05%)	Pseudomonadales (10.53%)	Moraxellaceae (8.68%)	*Arcobacter* (16.16%)
STMH-Aug	Proteobacteria (40.36%)	Betaproteobacteria (20.80%)	Flavobacteriales (19.96%)	Sporichthyaceae (19.67%)	*Flavobacterium* (13.41%)
	Bacteroidetes (25.24%)	Actinobacteria (20.76%)	Frankiales (19.67%)	Flavobacteriaceae (17.59%)	*Limnohabitans* (7.68%)
	Actinobacteria (22.65%)	Flavobacteria (19.96%)	Burkholderiales (14.76%)	Comamonadaceae (12.12%)	*Novosphingobium* (6.09%)
STMH-Sep	Actinobacteria (57.40%)	Actinobacteria (57.00%)	Corynebacteriales (56.71%)	Mycobacteriaceae (56.71%)	*Mycobacterium* (56.71%)
	Proteobacteria (39.01%)	Gammaproteobacteria (29.59%)	Xanthomonadales (16.84%)	Nevskiaceae (16.33%)	*Nevskia* (16.33%)
	Planctomycetes (0.61%)	Alphaproteobacteria (8.66%)	Legionellales (6.67%)	Legionellaceae (4.73%)	*Legionella* (4.23%)
STMH-Oct	Bacteroidetes (53.30%)	Flavobacteria (46.59%)	Flavobacteriales (46.59%)	Flavobacteriaceae (38.34%)	*Flavobacterium* (36.01%)
	Proteobacteria (41.25%)	Gammaproteobacteria (21.29%)	Pseudomonadales (15.30%)	Moraxellaceae (10.39%)	*Acinetobacter* (10.17%)
	Firmicutes (2.57%)	Betaproteobacteria (7.88%)	Campylobacterales (7.77%)	Cryomorphaceae (8.25%)	*Arcobacter* (7.73%)
XQR-Aug	Proteobacteria (84.55%)	Gammaproteobacteria (56.31%)	Pseudomonadales (47.98%)	Pseudomonadaceae (30.73%)	*Pseudomonas* (30.73%)
	Bacteroidetes (10.79%)	Betaproteobacteria (16.11%)	Burkholderiales (14.12%)	Moraxellaceae (17.25%)	*Acinetobacter* (17.14%)
	Firmicutes (3.99%)	Bacteroidia (7.82%)	Xanthomonadales (8.03%)	Comamonadaceae (13.54%)	*Stenotrophomonas* (7.93%)
XQR-Sep	Bacteroidetes (50.88%)	Flavobacteria (48.11%)	Flavobacteriales (48.12%)	Flavobacteriaceae (43.88%)	*Flavobacterium* (43.45%)
	Proteobacteria (38.52%)	Betaproteobacteria (18.51%)	Burkholderiales (14.55%)	Comamonadaceae (13.40%)	*Limnohabitans* (7.68%)
	Actinobacteria (5.84%)	Alphaproteobacteria (9.67%)	Frankiales (4.79%)	Sporichthyaceae (4.79%)	*Novosphingobium* (4.20%)
XQR-Oct	Proteobacteria (60.02%)	Betaproteobacteria (38.56%)	Burkholderiales (34.35%)	Comamonadaceae (21.76%)	*Limnohabitans* (10.79%)
	Bacteroidetes (18.33%)	Gammaproteobacteria (13.28%)	Pseudomonadales (11.96%)	Sporichthyaceae (11.86%)	*Polynucleobacter* (8.19%)
	Actinobacteria (12.84%)	Actinobacteria (12.69%)	Frankiales (11.89%)	Burkholderiaceae (8.37%)	*Acinetobacter* (7.53%)
