# Supplementary material for: Bacterial plasmid-mediated quinolone resistance genes in aquatic environments in China
Source: Sci Rep. 2017 Jan 17;7:40610. doi: 10.1038/srep40610 (PMC5240147; doi:10.1038/srep40610)
Supplement: Supplementary Information [file srep40610-s1.pdf]

Bacterial plasmid-mediated quinolone resistance genes in aquatic environments in China

Lei Yan [1], Dan Liu [1], Xin-Hua Wang [2], Yunkun Wang [2], Bo Zhang[1], Mingyu Wang [1, *], Hai Xu [1, *]

[1] State Key Laboratory of Microbial Technology, School of Life Sciences, Shandong University, Jinan 250100, China

[2] Shandong Provincial Key Laboratory of Water Pollution Control and Resource Reuse, School of Environmental Science and Engineering, Shandong University, Jinan 250100, China

* To whom correspondence is addressed: Hai Xu, haixu@sdu.edu.cn; Mingyu Wang, wangmingyu@sdu.edu.cn

Supplementary Table S1-S3

Supplementary Table S1. Combined correlation (all four sampling sites) between antimicrobial resistance genes. Significantly correlated genes are highlighted.

Supplementary Table S2. Correlation between antimicrobial resistance genes at four sampling sites. Significantly correlated genes are highlighted.

Supplementary Table S3. Primers used for qPCR reactions in this work.

Target gene	Primer sequence (5'-3')	Product size	Amplification efficiency	Source
qnrA	F:ATTTCTCACGCCAGGATTTG<br>R: CAGATCGGCATAGCTGAAG	158	1.001	1
qnrB	F: GGMATHGAAATTCGCCACTG<br>R: TTYGCBGYYCGCCAGTCGAA	245	0.950	1
qnrC	F: CAATGGCGAATTTCCAAG<br>R:ACCCGTAATGTAAGCAGAGC	139	0.952	This study
qnrD	F:GAGCTGATTTTCGAGGGGCTA<br>R:AGATCGGAGCCACGAAACAC	190	0.944	This study
qnrS	F: GACGTGCTAACTTGCGTGAT<br>R: TGGCATTGTTGGAAACTTG	118	1.046	1
aac(6')-Ib-cr	F: GCGTTTTAGCGCAAGAGTCC<br>R: GCCTTTGCCCAGTTGTGATG	179	1.032	This study
qepA	F：TGTGGATCGCCGCGTTTT<br>R：GCCAGCGTCAGCAGCATCA	124	1.002	This study
oqxA	F:GGGATAGTTTTAACGGTCGCATTG<br>R:TTCACGGGAGACGAGGTTGGT	266	1.048	2
oqxB	F:TCCTGATCTCCATTAACGCCCA<br>R:ACCGGAACCCATCTCGATGC	131	1.086	3
16S rDNA	F: CCCAGATGGGATTAGCTTGT<br>R: TCTGGACCGTGTCTCAGTTC	106	1.008	4
bla_{TEM}	F:GCKGCCAACTTACTTCTGACAACG<br>R:CTTTATCCGCCTCCATCCAGTCTA	247	1.055	5
Bla_{SHV}	F:GATGAACGCTTTCCCATGATG<br>R:CGCTGTTATCGCTCATGGTAA	214	1.023	6
$bla_{\mathrm{CTX\text{-}M}}$	F: ATTCCRGGCGAYCCGCGTGATACC<br>R:ACCGCGATATCGTTGGTGGTGCCAT	227	0.960	7

bla_{CMY}	F:CGTTAATCGCACCATCACC<br>R:CGTCTTACTAACCGATCCTAGC	172	0.933	8
bla_{DHA}	F:AACTTTCACAGGTGTGCTGGGT<br>R:GCTGCCACTGCTGATAGAA	218	1.033	9

References

1. Marti, E. & Balcázar, J. L. Real-time PCR assays for quantification of *qnr* genes in environmental water samples and chicken feces. *Appl. Environ. Microbiol.* **79,** 1743–1745 (2013).

2. Guo, W. *et al.* Development of quinoxaline 1, 4-dioxides resistance in *Escherichia coli* and molecular change under resistance selection. *PLoS ONE* **7**, e43322 (2012).

3. Li, J. *et al.* Plasmid-mediated quinolone resistance genes and antibiotic residues in wastewater and soil adjacent to swine feedlots: potential transfer to agricultural lands. *Environ. Health Perspect.* **120**, 1144-1149 (2012).

4. Kim, J. Y. & Lee, J. L. Multipurpose assessment for the quantification of *Vibrio* spp. and total bacteria in fish and seawater using multiplex real-time polymerase chain reaction. *J. Sci. Food Agric.* **94,** 2807–2817 (2014).

5. Xi, C. *et al.* Prevalence of antibiotic resistance in drinking water treatment and distribution systems. *Appl. Environ. Microbiol.* **75,** 5714–5718 (2009).

6. Kim, J. *et al.* Rapid detection of Extended Spectrum β-Lactamase (ESBL) for Enterobacteriaceae by use of a multiplex PCR-

based method. *Infect. Chemother.* **41**, 181 (2009).

7. Fujita, S. *et al.* Rapid identification of Gram-negative bacteria with and without CTX-M extended-spectrum β-lactamase from positive blood culture bottles by PCR followed by microchip gel electrophoresis. *J. Clin. Microbiol.* **49**, 1483-1488 (2011).

8. Kurpiel, P. M. & Hanson, N. D. Association of IS5 with divergent tandem $bla_{\text{CMY-2}}$ genes in clinical isolates of *Escherichia coli*. *J. Antimicrob. Chemother.* **66**, 1734-1738 (2011).

9. Brolund, A. *et al.* Development of a real-time SYBRGreen PCR assay for rapid detection of acquired *AmpC* in Enterobacteriaceae. *J. Microbiol. Methods* **82**, 229-233 (2010).
